# Tumor Location and Treatment Modality are Associated with Overall Survival in Adult Medulloblastoma

**DOI:** 10.7759/cureus.7061

**Published:** 2020-02-20

**Authors:** Anthony K Ma, Isaac Freedman, Jun Hui Lee, Danielle Miyagishima, Osama Ahmed, Jacky Yeung

**Affiliations:** 1 Neurological Surgery, Yale University, New Haven, USA; 2 Medicine, Yale University, New Haven, USA

**Keywords:** adult medulloblastoma, seer, outcome

## Abstract

Introduction

Medulloblastoma (MB) is an aggressive brain tumor most commonly found in children. Although prognostic factors are well studied in children, factors affecting survival in adults with medulloblastoma are unclear.

Methods

We queried the 1973-2015 United States Surveillance, Epidemiology, and End Results (SEER) registry to identify all adult cases of medulloblastoma, and performed multivariate survival analyses to assess the relationships amongst various clinical variables, including age, sex, race, tumor location, treatment modalities, and overall survival.

Results

A total of 857 patients, 20 years of age and older, with MB were identified in the SEER registry. Adult cases presented most frequently in the cerebellum (91.6%) compared to other less common regions (brain stem 3.2%, brain 2.2%, ventricle 1.8%). The overall median survival for adult MB is 60 months (SD = 94.3) and survival time is related to tumor location and course of treatment (P < 0.001). Multivariate Cox proportional hazard models showed that lesions found outside the cerebellum corresponded to worse median survival times (37 months) than those in the cerebellum (63 months) (hazard ratio 1.69, 95% CI 1.321-2.158, P = 0.001). Patients who were assigned chemotherapy had shorter survival (54 months) than those who were not (67 months) (HR 1.4515, 95% CI 1.26-1.671, P < 0.001), but receiving radiation therapy was associated with better overall survival (66 months) relative to not receiving radiation (25 months) (HR 0.581, 95% CI 0.48-0.70, P < 0.001).

Conclusions

Tumor location appears to be a significant prognostic factor for survival in adult MB. Recommended treatment regimes, likely reflective of the underlying aggressiveness of the tumor, also seem to impact survival.

## Introduction

Medulloblastoma (MB) is one of the most common primary tumors found in children with peak incidence between ages 15 to 19. It is, however, much less common in adult populations [1]. Multiple studies have pursued the question of whether MB is the same tumor across pediatric and adult populations. Giordana et al. suggested that there are differences in pathological features, such as nuclear polymorphism and histological signs of neuronal differentiation between pediatric and adult cases [2]. In addition to differences in pathologic features, more recent studies have shown genetic re-wirings that occur in promoting adult MB such as CDK6 amplification, 10q loss, and 17q gain, which were different from MYC/MYCN oncogene amplification that are typically represented in pediatric cases [3].

Given the differences in histological and underlying genetic aberrations, clinical studies have been done to assess the differences in prognostic factors and survival outcomes. Multiple studies have shown that MB occurs in children more frequently but has longer survival compared to adults [4]. Increasing age also has a negative correlation with survival outcomes [5]. Other demographic studies focusing on gender and race showed that gender has little association with survival outcomes in adulthood and race may be related to outcomes [6, 7].

Most studies assessing prognostic factors have focused on pediatric populations. Much less is known regarding survival in adult populations, given the rarity of this age demographic. Even less is known about how tumor location and treatment modalities are related to treatment outcomes. A recent smaller scale study done in China indicated that MB tumors in the fourth ventricular floor had a higher rate of recurrence [8]. An older larger scale study has shown that infratentorial lesions had distinct prognostic factors from supratentorial tumors [9].

In this study, we will perform a comprehensive analysis on the role of various prognostic indicators, such as tumor location and treatment modality, in survival in adult MB using the SEER database.

## Materials and methods

Data source and study cohort

All data used for our analysis was extracted from the US Surveillance, Epidemiology, and End Results database (SEER 1973-2014) which contains nearly 30% of cancer cases in the US population. We used the International Classification of Disease for Oncology (ICD-O-3) code for medulloblastoma (9470/3) and queried for adult patients with ages >= 19 to obtain our working dataset. Variables that we considered in this study included, age at diagnosis, gender, race, year of diagnosis, primary site of lesion, chemotherapy treatment status, radiation sequence with surgery, surgical history, and survival. All data obtained from SEER is public and therefore received exemption for review from the Yale Institutional Review Board (IRB).

Statistical analysis

Kaplan-Meier survival analysis was performed to assess the role of tumor location and treatment modalities on survival. We used the lifelines software package (http://lifelines.readthedocs.io/en/latest/) to perform univariate Cox proportional hazard models and multivariate Cox proportional hazard models to estimate the influence of each variable on survival. A threshold of p < 0.05 was used to define statistical significance.

## Results

Study population

In this study, we studied a total of 857 adult patients greater than the age of 19 with medulloblastoma diagnosed between the years 1973 and 2014. The median age of diagnosis was 31.0 years (SD 11.18), 58.5% of the cases were male, and 86.1% Caucasian (Table 1). The vast majority of all adult medulloblastoma tumors were found in the cerebellar region (91.6%), whereas non-cerebellar medulloblastomas were rare including the brain stem (3.2%), brain, NOS (2.2%), and ventricular regions (1.8%) (Table 2). The most common interventional treatment was surgical resection of the tumor with 95.1% of patients receiving surgery, 55.6% undergoing chemotherapy, and 79% receiving radiation therapy either before or after surgery (Table 3).

**Table 1:** Demographics of patients with adult medulloblastoma. Adult medulloblastoma predominantly presents in younger adults most commonly in the 20-24-year-old age group. This cancer type is more commonly found in males (58.5%) and Caucasians (86.1%).

	Adult Medulloblastoma (n = 857)
Age	
20-29	385 [44.9%]
30-39	273 [31.8%]
40-49	121 [14.1%]
50-59	54 [6.3%]
60-69	14 [1.6%]
70+	9 [1.0%]
Sex	
Female	356 [41.5%]
Male	501 [58.5%]
Race	
White	745 [86.1%]
Black	62 [7.2%]
Asian	49 [5.7%]
Other	10 [1.2%]

**Table 2:** Tumor locations of adult medulloblastoma. The majority of adult medulloblastoma presents in the cerebellum, whereas only 8.4% of tumors are found in non-cerebellar regions.

	Adult Medulloblastoma (n = 857)
C70.0-Cerebral meninges	0.1% (1)
C71.4-Occipital lobe	0.2% (2)
C71.0-Cerebrum	0.4% (3)
C72.0-Spinal cord	0.1% (1)
C71.5-Ventricle, NOS	1.8% (15)
C71.9-Brain, NOS	2.2% (19)
C71.6-Cerebellum, NOS	91.6% (785)
C71.8-Overlapping lesion of brain	0.2% (2)
C71.7-Brain stem, 3.2	3.2% (27)
C71.3-Parietal lobe	0.1% (1)
C71.1-Frontal lobe	0.1% (1)

**Table 3:** Treatment modalities for adult medulloblastoma. Common treatment modalities for medulloblastoma include surgical intervention in 95.1%, chemotherapy in 44.4%, and radiation before or after surgery in 79.0% of cases.

	Adult Medulloblastoma (n = 857)
Surgery	
Surgery performed	95.1% (815)
Surgery recommended but not performed	2.1% (18)
Not recommended	2.0% (17)
Unknown	0.5% (4)
Contraindicated to other condition	0.2% (2)
Patient refused	0.1% (1)
Chemo	
Yes	44.4% (384)
No/Unknown	55.6% (481)
Radiation	
Radiation after surgery	77.5% (670)
No radiation and/or cancer-directed surgery	20.3% (176)
Radiation prior to surgery	1.5% (13)
Sequence unknown, but both were given	0.3% (3)
Radiation before and after surgery	0.2% (2)
Intraoperative rad with other rad before/after surgery	0.1% (1)

Tumor location as related to overall survival

**Figure 1:**
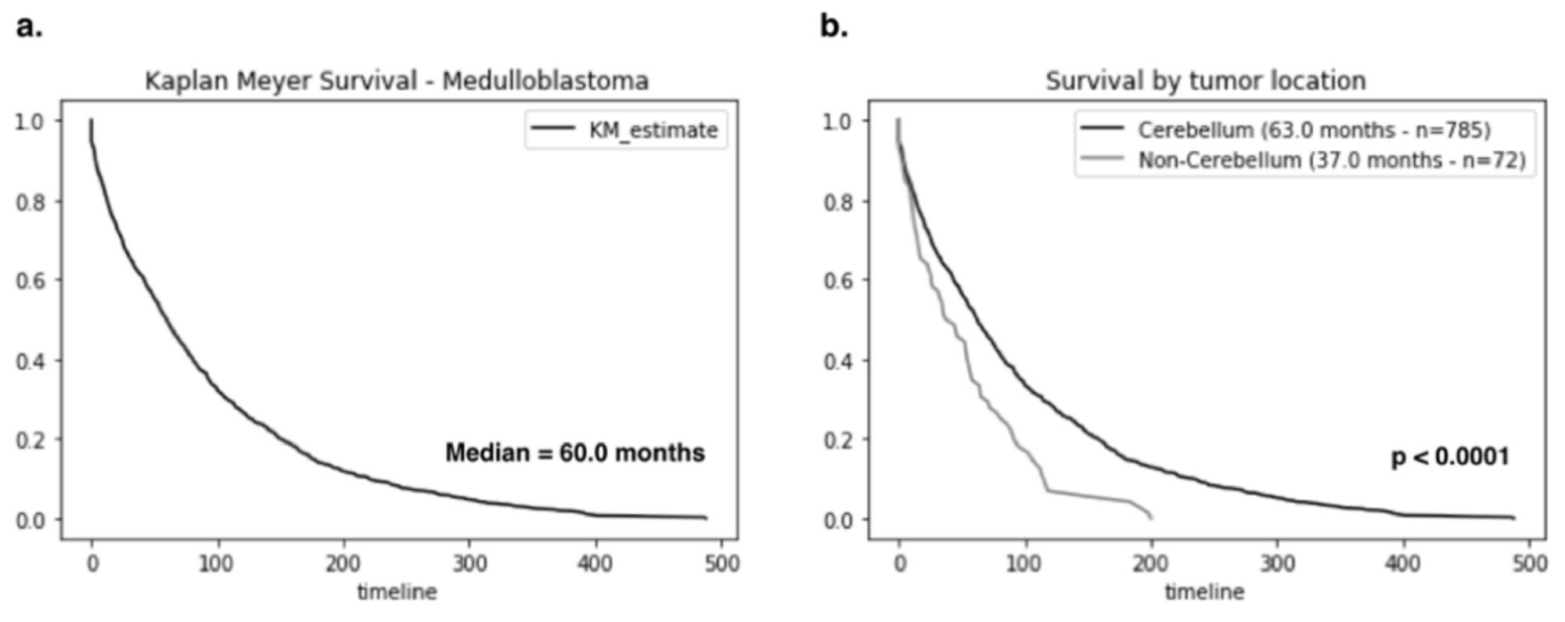
Overall survival of adult patients with medulloblastoma. (a) Kaplan-Meier survival curve for adult medulloblastoma with median survival of 60.0 months. (b) Tumor location is a significant predictor for survival as cerebellar medulloblastoma has median survival of 63.0 months (n = 785) while lesions found outside the cerebellum had survival time of only 37.0 months (n = 72) p < 0.0001.

To further profile the association between tumor location and survival time, we performed Kaplan-Meier survival analysis to our dataset. Amongst adult patients, the median survival time from time of diagnosis has a median of 60.0 months (Figure 1a). Of the 785 patients who had cerebellar medulloblastoma, the median survival time was 63.0 months which is slightly above the median for the entire cohort. The 72 patients who had non-cerebellar lesions (i.e., brainstem, ventricle, etc.) had median survival times of only 37.0 months. This drastic reduction in survival is apparent in the Kaplan-Meier survival curve and statistically significant (p < 0.001) (Figure 1b).

The role of treatment modality in survival outcomes

**Figure 2:**
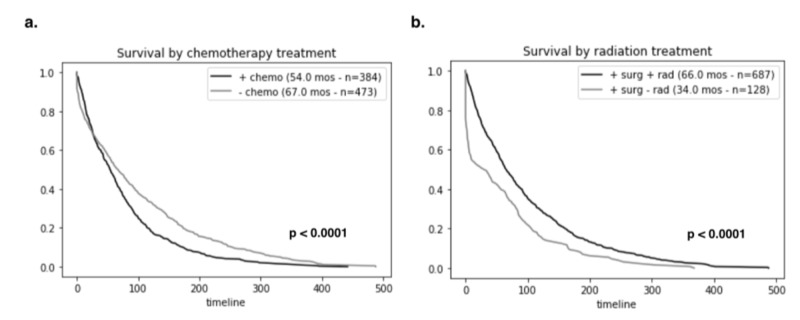
Overall survival of adult patients with medulloblastoma based on treatment modalities. (a) Patients who were highly recommended chemotherapy had shorter survival times of 54.0 months (n = 384) when compared to individuals who were not offered the same treatment (67.0 months, n = 473), suggesting the underlying aggressiveness of the tumor. (b) Those who received radiation before, after, or during surgery had much longer survival times (66.0 months, n = 687) as compared to those who had surgery without any radiation therapy (34.0 months, n = 128).

**Figure 3:**
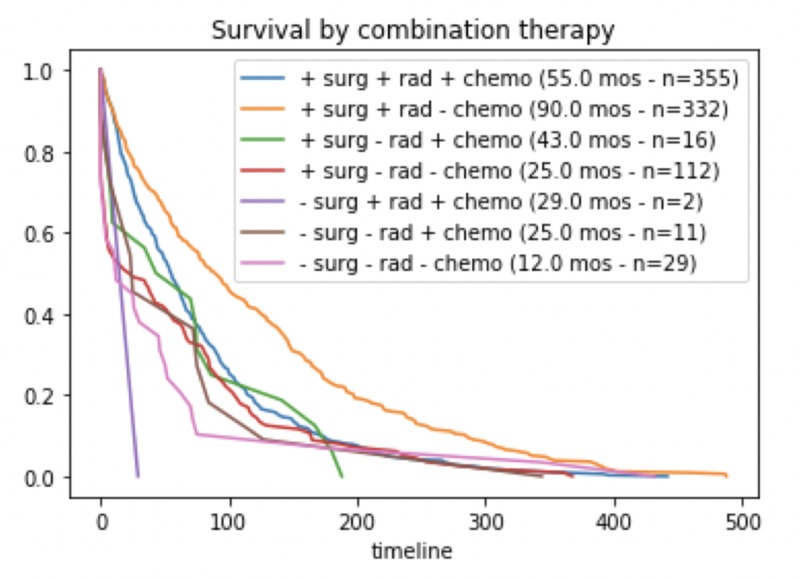
Overall survival based on different treatment modalities. Survival outcomes by different combinations of therapy involving surgery, radiation, and chemotherapy. Surgery serves as the mainstay treatment for medulloblastoma. Patients who require chemotherapy treatment had worse outcomes. Adding radiation therapy before, after, or during surgery significantly improves duration of survival.

We performed a series of Kaplan-Meier survival analyses on our patient cohort stratified by treatment interventions to analyze the role of treatment modality on survival outcomes. Out of the 384 patients who received chemotherapy, the median survival was 54.0 months as compared to 67.0 months in those that received no chemotherapy (p < 0.0001) (Figure 2a). Radiation, on the other hand, had a significant benefit to adult medulloblastoma patients. Out of the 687 patients who received radiation before, during, or after surgery, the median survival time was 66.0 months which is 6.0 months longer than the overall median. Patients who only had surgery but did not receive, refused, or were contraindicated with radiation therapy only had a survival time of 34.0 months (Figure 2b). Overall, radiation treatment in addition to surgical resection is associated with longer survival (Figure 3).

Multivariate analysis of prognostic factors that predict survival in adult medulloblastoma

To assess the association between predictor variables of interest in determining survival outcomes, we created a multivariate Cox proportional hazards model. From our analysis, we see that age of diagnosis has minor, but significant, influence over survival outcomes (HR 1.015, CI 1.009-1.020 p < 0.001). Finally, surgical intervention alone does not seem to have strong correlations with either improved or worsened outcomes. Interestingly, medulloblastomas that were found outside the cerebellum had a significantly worse outcome than those found inside the cerebellum (HR 1.689, CI 1.260-1.671, p < 0.001). Patients who were recommended and received chemotherapy adjuvant to surgery were also amongst those who had shorter survival times (HR 1.451, CI 1.260-1.671, p < 0.001). Radiation therapy either before or after surgical intervention, however, does seem to lead to significant improvements in survival for patients (HR 0.581, CI 0.479-0.703, p < 0.001) (Table 4).

**Table 4:** Multivariate Cox proportional hazard model. When performing a multivariate Cox proportional hazard model, we identified primary tumor location, chemotherapy, and radiation treatment to be prognostic factors that have significant influence on survival duration.

Covariate	HR (95% CI)	p-value
Age at diagnosis	1.02 (1.01, 1.02)	<0.001*
Sex	1.03 (0.90, 1.18)	0.703
Primary site	1.69 (1.32, 2.16)	<0.001*
Chemotherapy	1.45 (1.26, 1.67)	<0.001*
Radiation	0.58 (0.48, 0.70)	<0.001*
Surgery	1.00 (0.71, 1.42)	0.987

## Discussion

Many studies have shown that the underlying genetic and pathological progression for MB may be different in adults. For example, a large scale genome wide association study identified more passenger mutations linked with adult MB than in children [10]. Genetic rewirings such as CDK6 amplification, 10q loss, and 17q gain that promote MB in adults are shown to be different from the MYC oncogene amplification that is found in pediatric cases [3, 10]. Histological features such as nuclear polymorphisms were much more frequent in children as compared to GFAP-positive tumor cells that were sensitive markers for adult MB [2]. Given the inherent differences between pediatric and adult MB, it is important to investigate the predictive factors for survival in both populations.

Our analysis of adult medulloblastoma patients demonstrated multiple key findings that were not yet explored in adult populations in MB. Our study corroborates with previously published literature that increasing age is correlated with declining survival rates [5]. Gender, however, does not have a statistically significant association with survival in adults as compared to previous studies that point to better outcomes in female infants [6]. Furthermore, we demonstrated that tumor location is strongly associated with survival for adult MB patients, with those having cerebellar lesions have much better outcomes than those with lesions outside the cerebellum. It has been shown that medulloblastomas appear at higher frequency in lateral cerebellar locations and have better outcomes than tumors in the supratentorial region [9]. We found that when further stratifying MB by location, that lesions found in the cerebellum had better outcomes than rarer MB tumors found in other infratentorial locations such as the brain stem or ventricles. In terms of treatment modality, a recent study by Weil et al. demonstrated that radiation and surgery independently predict better survival outcome for children [11]. The influences of these treatments were not extensively studied in adults.

We found that treatment modalities like chemotherapy and radiation had differing associations on survival outcomes for adult MB patients. Patients who received chemotherapy seemed to have more severe progression of the disease and had worse outcomes. This may be confounded by the possibility that patients who were administered chemotherapy were more likely to have progressive disease. Conversely, receiving radiation either before, during, or after surgery had positive influence on survival. Recently, Beier et al. demonstrated in NOA-07, a pilot study of radiochemotherapy as first-line treatment, that radio-polychemotherapy led to considerable toxicity [12]. Prospective studies are needed to characterize the optimal treatments for these patients as there is a paucity of evidence to support any standardized treatment regimen [13].

A few caveats must be kept in mind in addition to the inherent retrospective nature of this study using the SEER database. First, in our analysis of tumor location as a predictive marker for survival we found that there were many more cases of cerebellar MB (n = 785) than non-cerebellar lesions (n = 72). Having such imbalances our dataset may influence the power of our statistical conclusions. We faced a similar situation when comparing the efficacy of receiving radiation (n = 683) compared to no radiation (n = 168). Furthermore, our results show that patients who received chemotherapy in addition to surgery actually had shorter survival times than those who were not recommended chemotherapy. The paradoxical shorter survival for those who received chemotherapy compared to those who did not is statistically significant and may indicate the underlying aggressiveness of the tumor that would prompt physicians to give chemotherapy potentially for measures of palliation rather than treatment. Lastly, the SEER database does not account for genetic diagnostics and heterogenous nature of different radiation modalities and chemotherapies inherent in such a database.

## Conclusions

We present the most comprehensive study to date on adult medulloblastoma in studying the role of tumor locality and treatment modality in survival outcomes. This retrospective study demonstrated that MBs in non-cerebellar regions were highly correlated with worse outcomes. Furthermore, being recommended chemotherapy was linked to shorter survival times as it may indicate the greater severity of the underlying disease. Finally, receiving radiation therapy seems to be associated with longer survival times.
